# Supplementary material for: Cortico-basal white matter alterations occurring in Parkinson’s disease
Source: PLoS One. 2019 Aug 19;14(8):e0214343. doi: 10.1371/journal.pone.0214343 (PMC6699705; doi:10.1371/journal.pone.0214343)
Supplement: S1 File — Fig A. STN Atlases. Subthalamic nucleus (STN) atlas in MNI152 1mm space where the Parkinson’s disease (PD) STN is in purple, and the healthy control (HC) in orange. The first control analysis is shown where the group specific atlases were switched so that the PD STN was registered to each HC, and vice versa. The last image shows the second control analysis where spheres were derived from the group specific center of gravity coordinates and expanded by 4.5mm. Table A. Tract strength descriptive per tract, per group, for the first control analysis which switches group specific atlases. Fig B. Tract Strengths: First Control Analysis. Tract strengths for the first control analysis, collapsed across hemisphere per structure, with healthy control (HC) subjects in orange and Parkinson’s disease (PD) patients in purple. Table B. Tract strength descriptive per tract, per group, for the second control analysis which uses spherical ROIs as an atlas. Fig C. Tract Strengths: Second Control Analysis. Tract strengths for the second control analysis, collapsed across hemisphere per structure, with healthy control (HC) subjects in orange and Parkinson’s disease (PD) patients in purple. Table C. Diffusion Tensor Imaging (DTI) descriptive statistics of axial diffusivity, fractional anisotropy and mean diffusivity per tract for the first control analysis. Table D. Diffusion Tensor Imaging (DTI) descriptive statistics of axial diffusivity, fractional anisotropy and mean diffusivity per tract, per group for the second control analysis. (DOCX) [file pone.0214343.s001.docx]

**Supporting information**

**S1 File : Supporting information : Control analysis**

**Rational and Method**

Two additional sets of control analyses were conducted to assess whether any findings from the initial analyses were due the use of age and disease specific atlases of the STN, which differ in both volume and location. Two additional sets of control analyses were conducted to assess whether any findings from the initial analyses were due the use of age and disease specific atlases of the STN, which differ in both volume and location. For the first control analysis, the group specific masks were interchanged, so that PD STN atlas was registered from standard MNI152 T1 1mm space to each individual healthy control subject, and the elderly subject STN atlas was registered from MNI to the individual space of each PD patient with a nonlinear transform. The first control analysis allows us to test whether the initial results are due to group differences in both volume and location of the probability atlas. For the second control analysis, a spherical region of interest was created in MNI space with a diameter of 9mm and resulted in a sphere volume of 383.88mm^3^ (Fig A). The 9mm diameter corresponds to the average length of the healthy STN ^[1]^. The location of the sphere was based on the CoG of the group specific probabilistic STN atlases. Subsequently, spheres were binarized and linearly registered to individual b0 space for each individual using the previously obtained transformation matrices. The second control analysis allows us to test whether the initial results are due to group differences in volume while taking the disease specific changes in location into account.

**Fig A: STN Atlases**

**
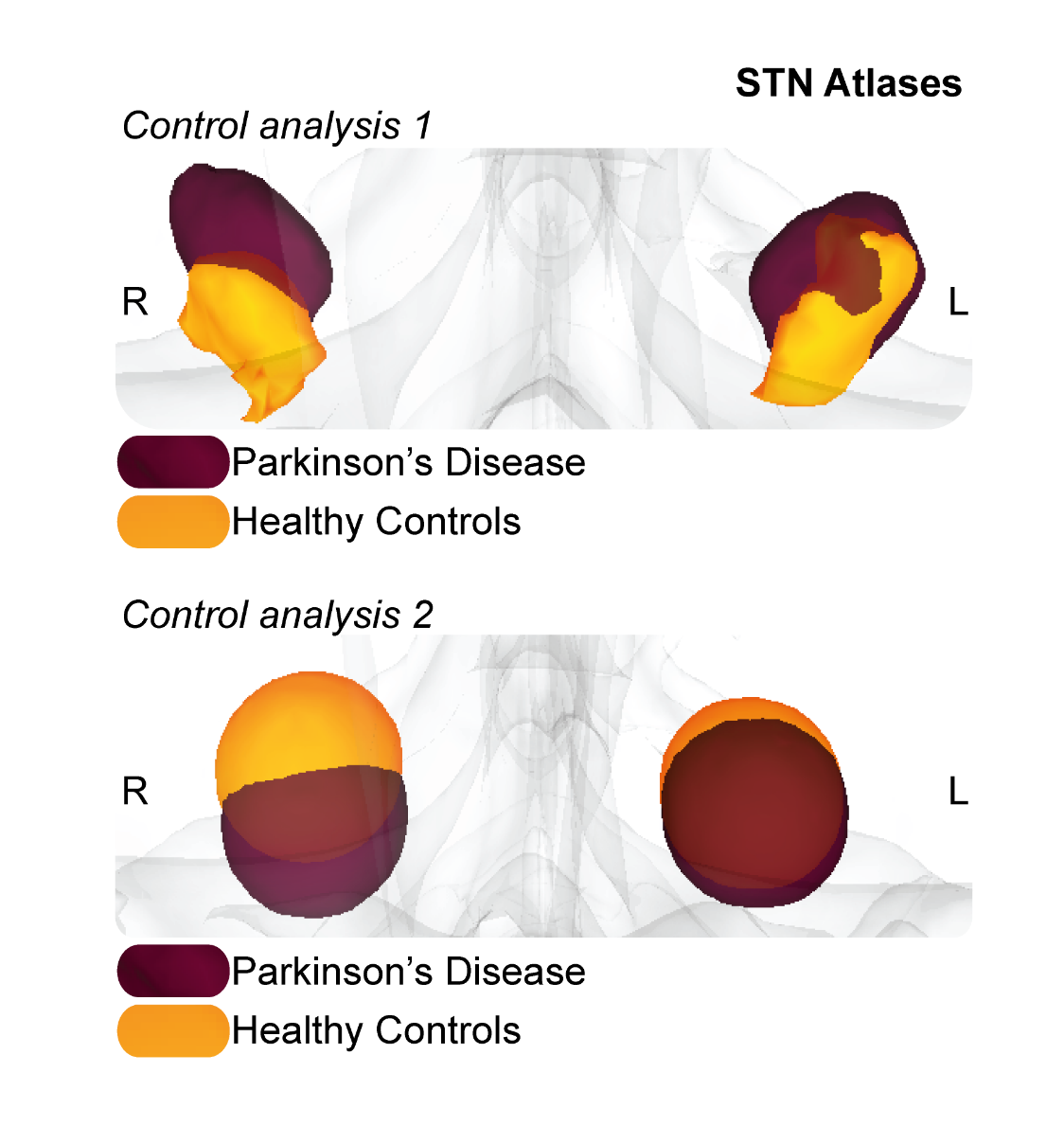
**

Subthalamic nucleus (STN) atlas in MNI152 1mm space where the Parkinson’s disease (PD) STN is in purple, and the healthy control (HC) in orange. The first control analysis is shown where the group specific atlases were switched so that the PD STN was registered to each HC, and vice versa. The last image shows the second control analysis where spheres were derived from the group specific center of gravity coordinates and expanded by 4.5mm*.*

**Probabilistic tractography**

Identical probtrackX parameters were applied to each set of STN atlases (original, first control analysis, and second control analysis).

**Results**

The statistical approach for the control analysis were conducted in the same manner as the initial analysis.

**Tract strengths**

For the first control analysis, the models including a main effect of structure, structure and group, as well as an interaction all provide decisive evidence for the alternative, against the null (all BF_10_ = > 100, ± < 1.7%). Though the model containing a main effect of only group provides moderate evidence for the null (BF_10_ = 0.13, ± 1.12%). The winning model, including an interaction between group and structure was 1325122 times more likely than the next largest model, with a main effect of structure, which according to the interpretation is decisive evidence for the superiority of the interaction over the main effect. See Fig B and Table A for descriptive statistics.

Additional post-hoc Bayesian t-tests were conducted to assess which connectivity profiles were driving the structure by group interaction. Substantial evidence was found for increased tract strengths between the STN and the ACC for healthy control subjects with a BF_10_ of 3.76. Decisive evidence was found for increased tract strengths between the STN and M1 for PD patients with a BF_10_ of 253.67 and for SMA with a BF_10_ of 18849.2.

For the second control analysis, the results were in line with the initial analysis. The main effect of structure, main effects of structure and group and the interaction all provide decisive evidence for the alternative, against the null (all BF_10_ = > 100, ± < 3%). The largest model included a main effect of both group and structure, which was 8 times more likely than the second largest model that included a main effect of only structure, and 89 times more likely than the interaction. See Fig C and Table B for descriptive statistics.

**Table A. Tract strength descriptive per tract, per group, for the first control analysis which switches group specific atlases**

| **Mean (S.D)** |
| --- |

| **Tract** | **HC** | **PD** |
| --- | --- | --- |
| **ACC** | 0.28 *(0.18)* | 0.20 (*0.17*) |
| **DLPFC** | 0.26 (*0.17*) | 0.21 (*0.16*) |
| **M1** | 0.38 (*0.15*) | 0.48 (*0.15*) |
| **Pre-SMA** | 0.59 (*0.11*) | 0.60 (*0.15*) |
| **SMA** | 0.54 (*0.14*) | 0.67 (*0.13*) |
| **POp** | 0.39 (*0.18*) | 0.37 (*0.22*) |

**Fig B: Tract Strengths­­­: First Control Analysis**

**
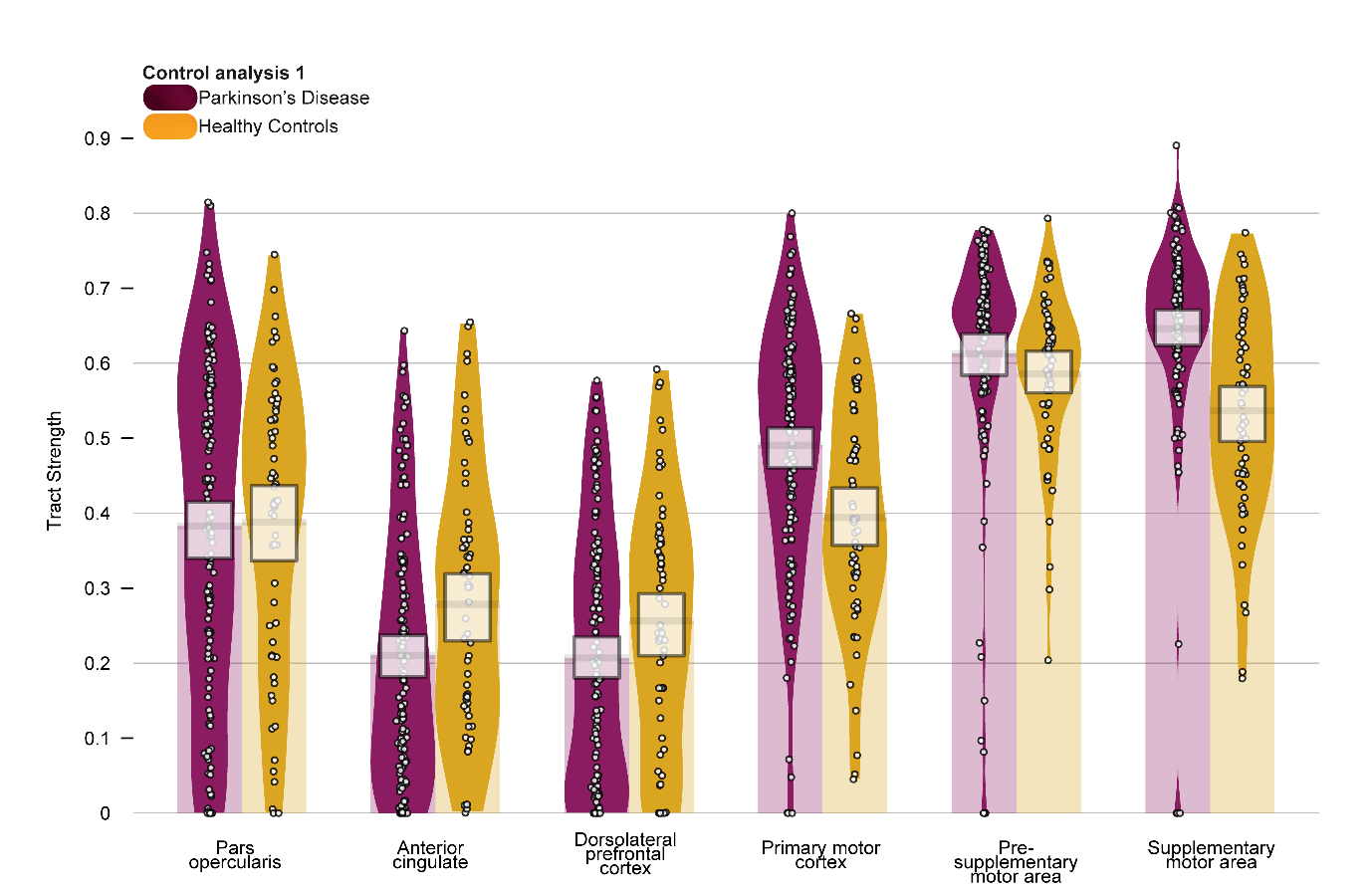
**

Tract strengths for the first control analysis, collapsed across hemisphere per structure, with healthy control (HC) subjects in orange and Parkinson’s disease (PD) patients in purple.

**Table B. Tract strength descriptive per tract, per group, for the second control analysis which uses spherical ROIs as an atlas**

| **Mean (S.D)** |
| --- |

| **Tract** | **HC** | **PD** |
| --- | --- | --- |
| **ACC** | 0.26 (*0.18*) | 0.19 (*0.12*) |
| **DLPFC** | 0.23 (*0.14*) | 0.18 (*0.11*) |
| **M1** | 0.54 (*0.14*) | 0.51 (*0.15*) |
| **Pre-SMA** | 0.58 (*0.11*) | 0.52 (*0.16*) |
| **SMA** | 0.60 (*0.11*) | 0.58 (*0.15*) |
| **POp** | 0.41 *(0.20)* | 0.36 (*0.19*) |

**Fig C: Tract Strengths­­­: Second Control Analysis**

**
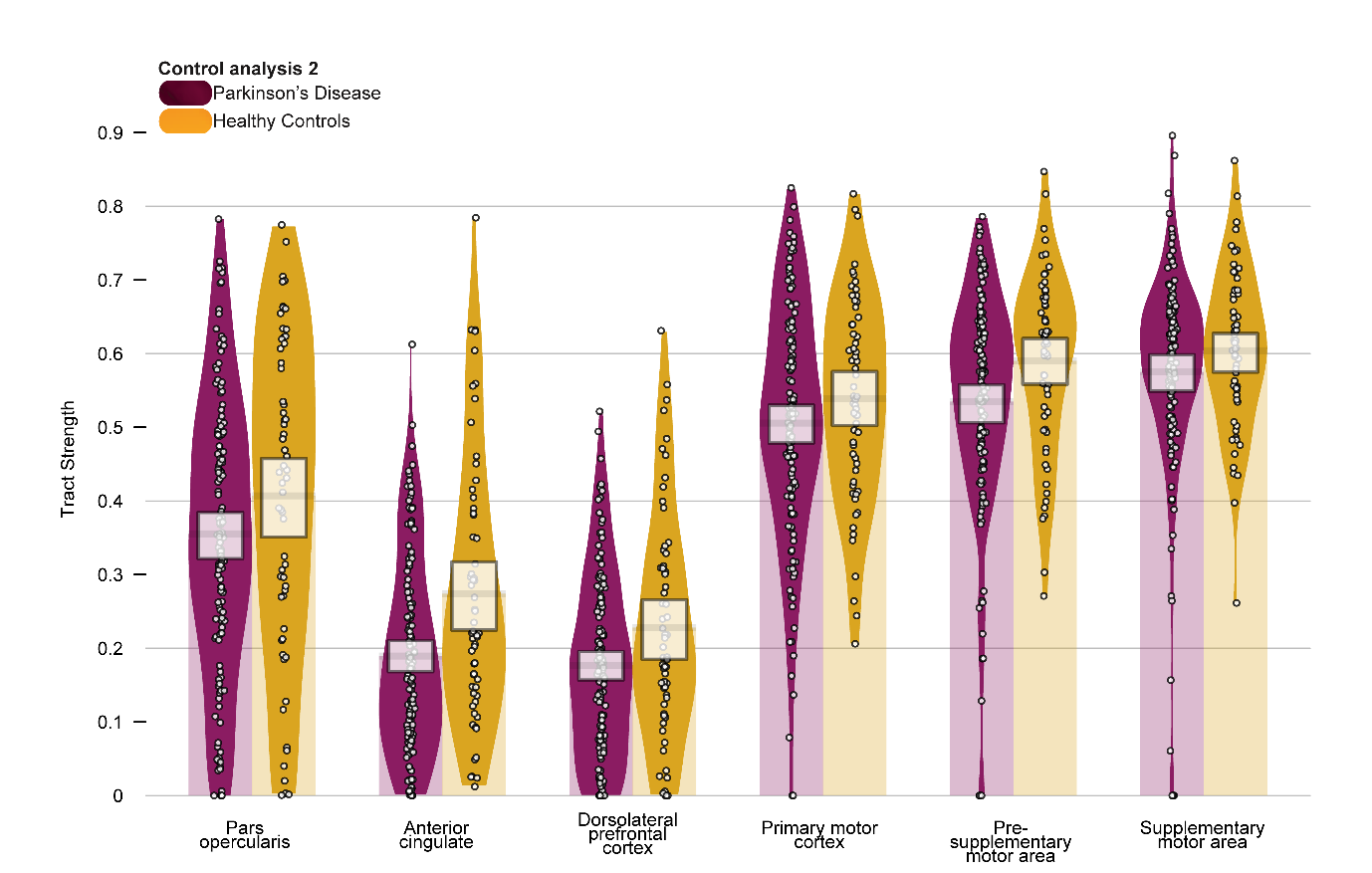
**

Tract strengths for the second control analysis, collapsed across hemisphere per structure, with healthy control (HC) subjects in orange and Parkinson’s disease (PD) patients in purple.

**DTI Metrics**

For the first control analysis for AD, the models including a main effect of structure, structure and group, as well as an interaction all provide decisive evidence for the alternative, against the null (all BF_10_ = > 100, ± < 2.5%). Though the model containing a main effect of only group provides anecdotal evidence for the alternative over the null (BF_10_ = 1.54, ± 2.6%). The winning model, including a main effect of both group and structure was 1.87 times more likely than the next largest model, with a main effect of structure, and 24.76 times more likely than the interaction, which provide anecdotal and strong evidence of the winning models superiority respectively.

For the first control analysis for FA, the models including a main effect of structure, structure and group, as well as an interaction all provide decisive evidence for the alternative, against the null (all BF_10_ = > 100, ± < 2.5%). Though the model containing a main effect of only group provides anecdotal evidence for the null (BF_01_ = 0.39, ± 3.3%). The winning model, including a main effect of structure was 1.71 times more likely than the next largest model, with a main effect of both group and structure, and 5.5 times more likely than the interaction, which provide anecdotal and moderate evidence of the winning models superiority respectively.

For the first control analysis for MD, the models including a main effect of structure, structure and group, as well as an interaction all provide decisive evidence for the alternative, against the null (all BF_10_ = > 100, ± < 3%). Though the model containing a main effect of only group provides anecdotal evidence for the alternative (BF_10_ = 1.58, ± 3%). The winning model, including a main effect of group and structure was twice as likely than the next largest model, with a main effect of only structure, and 18.13 times more likely than the interaction, which provide anecdotal and moderate evidence of the winning models superiority respectively.

For the second control analysis for AD, the models including a main effect of structure, structure and group, as well as an interaction all provide decisive evidence for the alternative, against the null (all BF_10_ = > 100, ± < 4.7%). Though the model containing a main effect of only group provides anecdotal evidence for the alternative over the null (BF_10_ = 1.47, ± 1.8%). The winning model, including a main effect of both group and structure was 1.79 times more likely than the next largest model, with a main effect of structure, and 23.12 times more likely than the interaction, which provide anecdotal and strong evidence of the winning models superiority respectively.

For the second control analysis for FA, the models including a main effect of structure, structure and group, as well as an interaction all provide decisive evidence for the alternative, against the null (all BF_10_ = > 100, ± < 2.5%). Though the model containing a main effect of only group provides anecdotal evidence for the null (BF_01_ = 0.44, ± 3.1%). The winning model, including a main effect of structure was 1.71 times more likely than the next largest model, with a main effect of both group and structure, and 5.6 times more likely than the interaction, which provide anecdotal and moderate evidence of the winning models superiority respectively.

For the second control analysis for MD, the models including a main effect of structure, structure and group, as well as an interaction all provide decisive evidence for the alternative, against the null (all BF_10_ = > 100, ± < 1.8%). Though the model containing a main effect of only group provides moderate evidence for the null (BF_01_ = 0.25, ± 1.4%). The winning model, including a main effect of structure was three times as likely than the next largest model, with a main effect of both group and structure, and 327 times more likely than the interaction, which provide moderate and decisive evidence of the winning models superiority respectively. See Tables C and D.

**Table C: Diffusion Tensor Imaging (DTI) descriptive statistics of axial diffusivity, fractional anisotropy and mean diffusivity per tract for the first control analysis**

| **Mead (S.D)** | | | | | | |
| --- | --- | --- | --- | --- | --- | --- |
| **Tract** | **AD** | | **FA** | | **MD** | |
|  | **HC** | **PD** | **HC** | **PD** | **HC** | **PD** |
| **ACC** | 1.2e-03  (*5.3e-05*) | 1.2e-03  (*4.4e-05*) | 0.38  (*0.03*) | 0.40  (*0.02*) | 8.43e-04  (*5.46e-05*) | 8.2e-04  (*5.2e-05*) |
| **DLPFC** | 1.2e-03  (*5.0e-05*) | 1.2e-03  (*5.3e-05*) | 0.33  (*0.01*) | 0.35  (*0.01*) | 8.73e-04  (*4.97e-05*) | 8.5e-04  (*5.4e-05*) |
| **M1** | 1.3e-03  (*9.0e-05*) | 1.3e-03  (*7.4e-05*) | 0.40  (*0.04*) | 0.40  (*0.04*) | 9.09e-04  (*9.99e-05*) | 8.7e-04  (*8.4e-05*) |
| **Pre-SMA** | 1.2e-03  (*5.4e-05*) | 1.2e-03  (*4.9e-05*) | 0.38  (*0.01*) | 0.38  (*0.03*) | 8.35e-04  (*6.08e-05*) | 8.1e-04  (*5.3e-05*) |
| **SMA** | 1.3e-03  (*7.4e-05*) | 1.3e-03  (*8.2e-05*) | 0.38  (*0.03*) | 0.37  (*0.02*) | 9.34e-04  (*7.94e-05*) | 9.2e-04  (*8.8e-05*) |
| **POp** | 1.3e-03  (*7.4e-05*) | 1.1e-03  (*5.8e-05*) | 0.33  (*0.02*) | 0.36  (*0.01*) | 9.64e-04  (*7.08e-05*) | 9.3e-04  (*5.5e-05*) |

**Table D: Diffusion Tensor Imaging (DTI) descriptive statistics of axial diffusivity, fractional anisotropy and mean diffusivity per tract, per group for the second control analysis***.*

| **Mean (S.D)** | | | | | | |
| --- | --- | --- | --- | --- | --- | --- |
| **Tract** | **AD** | | **FA** | | **MD** | |
|  | **HC** | **PD** | **HC** | **PD** | **HC** | **PD** |
| **ACC** | 1.1e-03  (*4.4e-05*) | 1.2e-03  (*4.0e-05*) | 0.43  (*0.03*) | 0.42  (*0.03*) | 8.2e-04  (*5.0e-05*) | 8.1e-04  (*4.6e-05*) |
| **DLPFC** | 1.1e-03  (*4.2e-05*) | 1.2e-03  (*4.9e-05*) | 0.39  (*0.01*) | 0.38  (*0.01*) | 8.4e-04  (*4.0e-05*) | 8.4e-04  (*4.7e-05*) |
| **M1** | 1.3e-03  (*7.2e-05*) | 1.1e-03  (*6.2e-05*) | 0.43  (*0.00*) | 0.42  (*0.02*) | 8.7e-04  (*8.0e-05*) | 8.5e-04  (*7.0e-05*) |
| **Pre-SMA** | 1.1e-03  (*4.9e-05*) | 1.2e-03  (*4.7e-05*) | 0.42  (*0.23*) | 0.42  (*0.01*) | 8.3e-04  (*5.4e-05*) | 8.2e-04  (*4.7e-05*) |
| **SMA** | 1.3e-03  (*6.0e-05*) | 1.3e-03  (*6.5e-05*) | 0.42  (*0.03*) | 0.42  (*0.03*) | 8.9e-04  (*6.4e-05*) | 8.9e-04  (*6.8e-05*) |
| **POp** | 1.3e-03  (*5.8e-05*) | 1.1e-03  (*5.1e-05*) | 0.39  (*0.02*) | 0.39  (*0.01*) | 9.0e-04  (*5.6e-05*) | 9.0e-04  (*4.6e-05*) |

**Conclusions on the effect of STN atlas**

To assess whether the use of separate probabilistic atlases for PD and for healthy controls produced more accurate results, we ran two sets of control analyses. For the first control analysis, interchanging the atlases across groups illustrated that failing to account for disease specific changes influences the results. When using the healthy control probabilistic atlas in the PD group we find that the STN-M1 and SMA tract strengths increased for PD relative to controls. From a clinical perspective, the finding of increased connectivity in PD seems erroneous. While it is plausible that functional MRI (fMRI) studies may find increased connectivity between the STN and motor cortices in PD due to either pathological hyperactivity of the STN or some compensatory mechanism ^[2]^, previous DWI studies have reported a general decrease in cortico-basal connectivity for PD patients, though not specific to the STN ^[3].^ Therefore, our results are better explained by a false positive, whereby using a healthy STN atlas for probabilistic tractography in PD fails to account for the pathological shift of the STN and accordingly tracks from an incorrect location. Similarly, the second control analysis supports the use of group specific atlases. Here, tracking from a sphere that accounts for changes in location but not variations in shape and volume produces a similar trend as the original analyses though with less certainty, as indexed by the decrease in BF values. In sum, the results of both the first and second control analysis, show that the use of group specific atlases are more accurate than using atlases that do not account for disease- and age-related structural alterations.

**References**

1. Baudrexel S, Witte T, Seifried C, von Wegner F, Beissner F, Klein JC, et al. Resting state fMRI reveals increased subthalamic nucleus-motor cortex connectivity in Parkinson’s disease. Neuroimage. 2011;
2. Forstmann BU, Isaacs BR, Temel Y. Ultra High Field MRI-Guided Deep Brain Stimulation. Trends Biotechnol [Internet]. 2017;35(10):904–7. Available from: http://dx.doi.org/10.1016/j.tibtech.2017.06.010
3. Cochrane CJ, Ebmeier KP. Diffusion tensor imaging in parkinsonian syndromes: A systematic review and meta-analysis. Neurology. 2013.
