# Supplementary material for: Cortico-basal white matter alterations occurring in Parkinson’s disease
Source: PLoS One. 2019 Aug 19;14(8):e0214343. doi: 10.1371/journal.pone.0214343 (PMC6699705; doi:10.1371/journal.pone.0214343)
Supplement: S2 File — Table A. Pearson’s Rho. Interpretation of Pearson’s Rho Correlation Coefficients[1]. Table B. Correlation between disease progression and tract strength. Table C. Correlation between medication response and tract strength. Table D. Correlation between disease progression and fractional anisotropy. Table E. Correlation between medication response and fractional anisotropy. (DOCX) [file pone.0214343.s002.docx]

**Supporting information**

**S2 File : Supporting information : Correlation results**

**Results : Correlations**

Bayesian paired correlations with a Pearson’s Rho correlation coefficient (Table A) was conducted to assess whether for each PD patient, disease progression or medication response correlated with either their tract strength or respective FA measures. Below, contralateral is used to refer to the contralateral hemisphere to side of symptom onset, and ipsilateral is used to refer to the ipsilateral hemisphere to side of symptom onset.

**Table A. Pearson’s Rho**

| **Coefficient, *r*** | | |
| --- | --- | --- |
| **Positive** | **Negative** | **Size of correlation** |
| **< 0** | **< 0** | zero |
| **0.1 to 0.3** | **-0.1 to -0.3** | Small |
| **0.3 to 0.5** | **-0.3 to -0.5** | Medium |
| **0.5 to 1.0** | **-0.5 to 1.0** | Large |

Interpretation of Pearson’s Rho Correlation Coefficients[1].

**Disease progression with tract strength**

All results reported substantial evidence for no correlation between tract strengths and disease progression (Table B).

**Table B: Correlation between disease progression and tract strength**

| **Tract** | **Contralateral** | | **Ipsilateral** | |
| --- | --- | --- | --- | --- |
|  | **Correlation** | **BF**_10_ | **Correlation** | **BF**_10_ |
| **ACC** | - 0.06 | 0.18 | - 0.03 | 0.16 |
| **DLPFC** | - 0.21 | 0.15 | - 0.04 | 0.17 |
| **M1** | 0.20 | 0.20 | - 0.06 | 0.18 |
| **Pre-SMA** | 0.00 | 0.15 | - 0.00 | 0.15 |
| **SMA** | 0.00 | 0.14 | - 0.03 | 0.16 |
| **Pop** | 0.06 | 0.18 | 0.15 | 0.30 |

**Medication response with tract strength**

All results reported anecdotal (ACC) or substantial (DLPFC, M1, pre-SMA, SMA, POp) evidence for no correlation between tract strengths and medication response (Table C).

**Table C: Correlation between medication response and tract strength**

| **Tract** | **Contralateral** | | **Ipsilateral** | |
| --- | --- | --- | --- | --- |
|  | **Correlation** | **BF**_10_ | **Correlation** | **BF**_10_ |
| **ACC** | - 0.21 | 0.63 | - 0.04 | 0.15 |
| **DLPFC** | - 0.10 | 0.20 | - 0.08 | 0.20 |
| **M1** | - 0.13 | 0.30 | 0.11 | 0.22 |
| **Pre-SMA** | 0.11 | 0.21 | 0.14 | 0.31 |
| **SMA** | 0.04 | 0.16 | 0.05 | 0.17 |
| **POp** | 0.00 | 0.15 | - 0.03 | 0.14 |

**Disease progression with FA**

The only averaged FA per tract to show strong evidence of a correlation with disease progression was the DLPFC ipsilateral score (r = 0.35, BF_10_ = 16.50). All other results reported either anecdotal or substantial evidence for no correlation between FA and disease progression (Table D).

**Table D: Correlation between disease progression and fractional anisotropy**

| **Tract** | **Contralateral** | | **Ipsilateral** | |
| --- | --- | --- | --- | --- |
|  | **Correlation** | **BF** | **Correlation** | **BF** |
| **ACC** | 0.13 | 0.28 | 0.20 | 0.71 |
| **DLPFC** | 0.20 | 0.61 | 0.35 | 16.50 |
| **M1** | - 0.03 | 0.14 | - 0.01 | 0.15 |
| **Pre-SMA** | 0.10 | 0.23 | 0.09 | 0.18 |
| **SMA** | - 0.03 | 0.14 | -0.00 | 0.15 |
| **POp** | 0.15 | 0.36 | 0.11 | 0.22 |

**Medication response with FA**

All results reported substantial evidence for no correlation between FA and medication response (Table E).

**Table E: Correlation between medication response and fractional anisotropy**

| **Tract** | **Contralateral** | | **Ipsilateral** | |
| --- | --- | --- | --- | --- |
|  | **Correlation** | **BF** | **Correlation** | **BF** |
| **ACC** | 0.13 | 0.25 | 0.02 | 0.14 |
| **DLPFC** | 0.10 | 0.21 | 0.02 | 0.14 |
| **M1** | 0.04 | 0.16 | 0.09 | 0.19 |
| **Pre-SMA** | 0.02 | 0.24 | 0.11 | 0.22 |
| **SMA** | 0.00 | 0.15 | 0.03 | 0.16 |
| **POp** | 0.06 | 0.17 | - 0.03 | 0.16 |

**References**

1. Lambdin C. Significance tests as sorcery: Science is empirical—significance tests are not. Theory Psychol. 2012 Feb 25;22(1):67–90.
